# Dysfunction of the neurovascular unit in brain aging

**DOI:** 10.7555/JBR.36.20220105

**Published:** 2023-04-15

**Authors:** Shu Liu, Xu Yang, Fei Chen, Zhiyou Cai

**Affiliations:** 1 Chongqing Medical University, Chongqing 400042, China; 2 Chongqing Institute Green and Intelligent Technology, Chinese Academy of Sciences, Chongqing 400714, China; 3 Chongqing School, University of Chinese Academy of Sciences, Chongqing 400714, China; 4 Department of Neurology, Chongqing General Hospital, Chongqing 400013, China; 5 Chongqing Key Laboratory of Neurodegenerative Diseases, Chongqing 400013, China

**Keywords:** aging, neurovascular unit, Alzheimer's disease, Parkinson's disease

## Abstract

An emerging concept termed the neurovascular unit (NVU) underlines neurovascular coupling. It has been reported that NVU impairment can result in neurodegenerative diseases, such as Alzheimer's disease and Parkinson's disease. Aging is a complex and irreversible process caused by programmed and damage-related factors. Loss of biological functions and increased susceptibility to additional neurodegenerative diseases are major characteristics of aging. In this review, we describe the basics of the NVU and discuss the effect of aging on NVU basics. Furthermore, we summarize the mechanisms that increase NVU susceptibility to neurodegenerative diseases, such as Alzheimer's disease and Parkinson's disease. Finally, we discuss new treatments for neurodegenerative diseases and methods of maintaining an intact NVU that may delay or diminish aging.

## Introduction

The definition of aging refers to a time-dependent functional decline that affects most living organisms and involves a progressive loss of physiological integrity^[1]^. The aging population was 18% in 2000 and is projected to reach 38% by 2050. Studies also suggest that the number of people aged 60 or over will surpass that of adolescents aged 10–24 (*i.e.*, 2.1 billion versus 2.0 billion) by 2050^[2]^, which has implications for public health and finite resources. Given current trends, we are likely to see a rapid increase in the global prevalence of age-related diseases, specifically Alzheimer's disease (AD) and Parkinson's disease (PD)^[3]^. Therefore, as a global community, we must combine our resources to ensure that aging populations are healthier and can maintain good mental health as they age.

**Figure 1:**
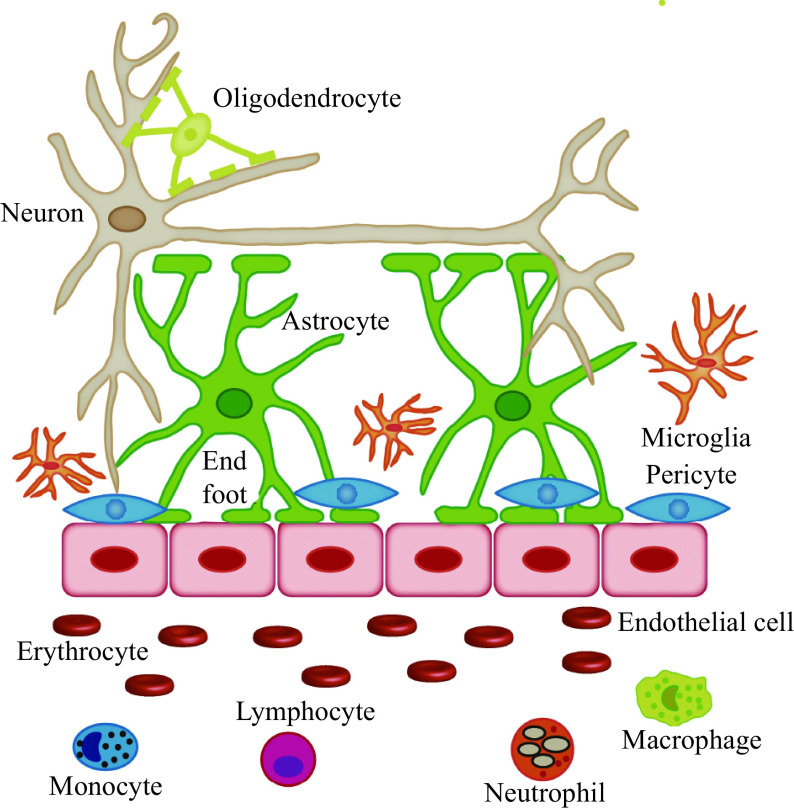
Structure schematic of young neurovascular unit.

Brain tissues are primarily composed of postmitotic cells and are particularly vulnerable to the effects of aging. The neurovascular unit (NVU) consists of a network of neurons, glial cells (*e.g.*, microglia, astrocytes, and oligodendrocytes), and vascular cells that include pericytes, endothelial cells (ECs), and smooth muscle cells. Capillary ECs are the main cellular components of NVUs, and junctional proteins create a barrier between the adjacent brain ECs^[4]^. Pericytes in the brain contact with ECs. The end-feet of astrocytes cover over 99% of cerebral capillaries to cause vital cell-cell and cell-neuron interactions. The coordination of these cells in the brain then modulates and regulates NVU characteristics^[5]^. Please see ***Fig. 1*** for a diagrammatic representation.

Recently, a large number of investigations indicate that brain aging may lead to dysfunctions of the NVU components, and the impaired NVU is closely related to neurodegenerative diseases. Therefore, this review outlines the basics of NVU and contemplates the effect of aging on NVU components. Furthermore, we discuss NVU impairment and aging-related neurodegenerative diseases, such as AD and PD. Towards the end of the article, we introduce some novel targets for therapeutic intervention to maintain NVU integration during aging, although further research is required. Ultimately, we hope that this traditional review will generate more researchable questions about how we manage neurodegenerative diseases in the future.

## Basics of neurovascular unit

### Glial cells

As a specialized glial cell, astrocytes outnumber neurons by over fivefold. Astrocytes actively maintain the homeostasis of central nervous system (CNS) by influencing the pH, equilibrium of ions and water, plasticity of neurotransmitters and synapses, and cerebral blood flow (CBF). Astrocytes are closely connected to neurons and have a 'cross-talk' with oligodendrocytes, which occurs *via* cell-cell contact and the secretion of cytokines, chemokines, exosomes, and signaling molecules^[6]^.

Microglia are the brain's immune cells and act as the first barrier to protect the CNS from pathogen invasion and to remove cellular debris^[7]^. Microglia in a resting state present ramified morphologies^[8]^; however, if there is an acute injury, microglia convert to an activated or reactive state and secrete a large array of inflammatory mediators, including cytokines (interleukin-1 [IL-1], IL-6, IL-12, IL-23, and tumor necrosis factor-alpha [TNF-α]), chemokines (CC motif chemokine ligand 2 [CCL2], C-X3-C motif chemokine ligand 1 [CX3CL1], and macrophage inflammatory protein 1 [MIP-1], proteases (matrix metalloproteinases [MMPs]), and free radicals^[9]^. This expression profile has been typically described as an 'M1-like' phenotype that can last for weeks or even months. After this period, their phenotypic characteristics are considered an 'M2-like' phenotype that affects the phagocytic, immunomodulatory, and anti-inflammatory processes^[10]^. Microglia are highly reactive immune cells in the brain that can alter their morphology and phenotypic characteristics in response to complex environmental changes.

Oligodendrocytes are myelinating cells with a high metabolic rate in the CNS. They produce myelin that is important for action potentials^[11]^. Furthermore, oligodendrocytes provide trophic support to axons, which is crucial for neuronal functionality^[12]^. Oligodendrocytes communicate with microglia by producing immunomodulatory factors, expressing receptors, and participating in the process of immunomodulatory in a complex way^[13]^.

### Neurons

Neurons, as the basic unit of the nervous system, serve in signal transmission, communication, and adjustment of the blood-brain barrier (BBB)^[14]^. Dendrites and axons in the brain make connections between neurons. In addition, neurons connect with different components of the NVU, such as glial cells and vascular cells, to maintain normal brain functions. Astrocytes protect neurons by releasing neurotrophic factors, producing antioxidants, and disposing of neuronal waste products^[15]^. The end-feet of astrocytes contact with presynaptic and postsynaptic neurons, forming the tripartite synapses that serve as bridges connecting neurons and microvessels^[16]^.

### Vascular cells

Vascular cells in the NVU include pericytes, ECs, and smooth muscle cells. In the brain, the proportion of pericytes to ECs was the highest, highlighting their importance within the NVU^[17]^. Pericytes, the isolated contractile cells on capillaries, are enclosed in the capillary wall and contact with ECs directly^[18]^. Although pericytes were previously considered as a structural component, recent studies have verified their multiple functions in the CNS^[19]^, including their active role in regulating angiogenesis^[20]^. Vascular remodeling requires vascular ECs to degrade basement membranes. Additionally, vascular ECs can secrete MMPs to increase the production of angiogenic factors, such as vascular endothelial growth factor (VEGF), to promote angiogenesis^[21]^. The activated pericytes also produce MMPs, including MMP2, MMP3, and MMP9, to degrade the basement membrane. Thus, the degraded basement membrane enhances BBB permeability^[22]^.

Pericytes are important for maintaining CNS homeostasis by endocytosis. For example, pericytes can clear amyloid-beta (Aβ) peptides in AD^[17]^. Moreover, pericytes regulate the maintenance of neuronal health by secreting neurotrophic factors, such as nerve growth factor (NGF) and brain-derived neurotrophic factor (BDNF). Recent studies have reported that pericytes produce Lama2 to help oligodendrocyte precursor cell differentiate into mature oligodendrocytes^[23]^.

In addition, the emerging role of pericytes in neuroinflammation has been observed. Pericytes secrete factors, such as chemokines (CCL2, CXCL1, and CXCL8) to upregulate intercellular adhesion molecule-1 (ICAM-1) and vascular cellular adhesion molecule-1 (VCAM-1) adhesion molecules on ECs, and these help the recruitment of peripheral immune cells, including monocytes, T and B cells, and lymphocytes, to the CNS parenchyma. The pericyte-mediated neuroinflammation has been suggested as a potential therapeutic target for the treatment of a range of brain disorders^[22,24]^. Furthermore, pericytes respond to inflammatory stimuli, such as LPS, TNF-α, IL-1β, and IFN-γ, and then produce nitric oxide (NO), MMPs as well as reactive oxygen species (ROS) to activate microglia and astrocytes^[25]^. In short, pericytes have a regulatory function, while supporting regeneration and acting as an anti-inflammatory in the brain.

## The effect of aging on the components of the neurovascular unit

### The effect of aging on neurons

Neuronal morphology rearranges during aging. Primary alterations of age-related neuronal structure involve degenerated dendritic trees and decreased dendritic shafts with the loss of various dendritic spines^[26]^. A study simulated the effects of dendritic pruning on neuronal morphology during aging, and characterized these dynamic implications using detailed computational modeling methods. The results have shown that neuronal integrity is broken down by dendritic pruning, and the firing rate is reduced, causing a reduction in energy consumption, energy efficiency, and dynamic range^[27]^. In addition, during brain aging, neuronal axons not only degrade but also decrease in number^[28]^.

Neuronal membranes are susceptible to oxidative stress due to their high lipid content^[26]^. Neurons accumulate the damaged and aggregated proteins during brain aging, resulting in oxidative stress that manifests in the accumulation of ROS production and the decrease of antioxidant capacity^[29]^. Some signal pathways can be activated to counteract the accumulation of ROS and reactive nitrogen species, such as the Nrf2 pathway; however, the Nrf2 activation is significantly lower in aged animals than in younger ones^[30]^. The abundant ROS production may induce neuroinflammation and neuronal death^[31]^.

Some organelles, including the endoplasmic reticulum, mitochondria, nucleus, and peroxisomes, are mainly distributed in the axons and dendrites of neurons. Mitochondria are the principal source of ROS in neuronal cells, and their structure does not change during brain aging. However, mtDNA mutations in damaged mitochondria are crucial for aging^[32]^. During brain aging, oxidative stress modifies endoplasmic reticulum homeostasis by triggering reticulum stress, increasing misfolded proteins, and upregulating the unfolded protein response. Peroxisomes are involved in many metabolic pathways and redox homeostasis to maintain the balance of brain metabolism, and they are closely related to cell aging, because of their susceptibility to ROS production. Peroxisomes decrease metabolic activity and catalase release to the cytosol during aging, and catalase levels have been reported to limit cellular senescence. These organelles aforementioned are related to the ATP generation, which is essential to maintain the stabilization of electrochemical neurotransmission and contribute to cell repair^[33]^. During aging, neurons are unable to generate sufficient ATP to sustain brain activity; therefore, synapses tend to alter and degenerate, eventually altering synaptic neurotransmission^[34]^.

**Figure 2:**
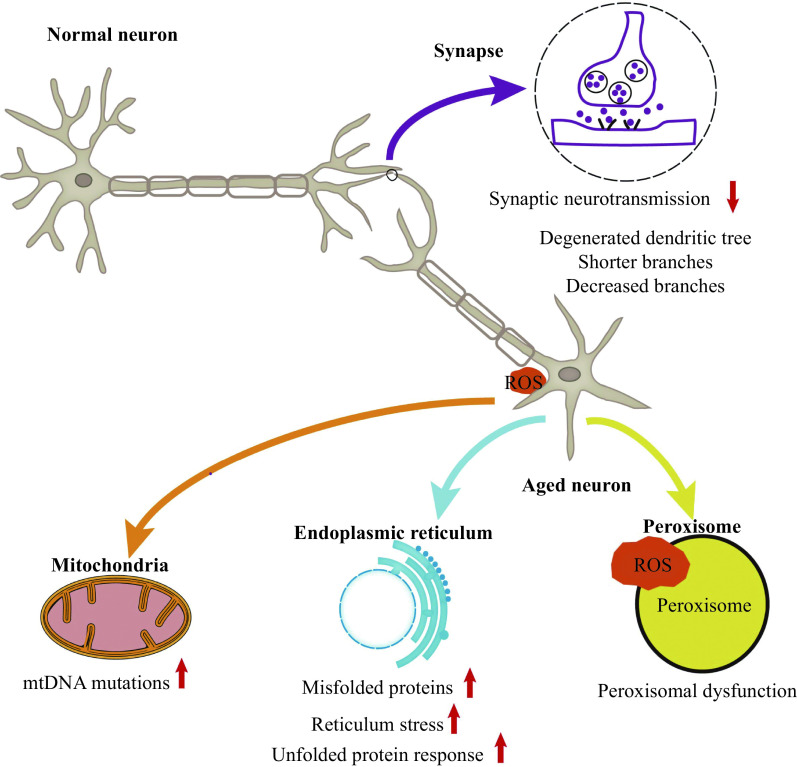
Impact of aging on neurons.

The brain constitutes only 2% of a person's body weight; however, it consumes approximately over 20% of the body's energy^[35]^. Different cell types have different ways of glucose metabolism in the brain. A large number of studies have shown that the energy metabolism of astrocytes is important to support the high energy requirement of neurons^[36–37]^. Astrocytes cultured *in vitro* produce large amounts of lactate, but neurons produce less lactate from glucose under a normal (21%) oxygen environment^[38]^, which implies that astrocytes take up glucose from microvessels and produce lactate as a supplement to neurons. In normal circumstances, Glucose transporter1 (GLUT1) transports glucose across the endothelium into astrocytes, then brain glucose is transferred into neurons by GLUT3 and GLUT4, forming a complex metabolic process. Astrocytes generate ATP and lactate through anaerobic glycolysis^[36]^. Consequently, lactate is released from astrocytes and is taken up by neighboring neurons through monocarboxylate transporters, which is called the astrocyte-neuron lactate shuttle^[39]^. Besides, GLUT3 enables the importation of glucose from blood vessels into neurons directly^[37]^. Thus, astrocytes are probably responsible for most of the energy supplementation of neurons, because 99% of the surface of microvessels is covered by astrocyte endfeet^[40]^. For example, the reduced glucose uptake and the decreased neuronal GLUTs have been reported in the brains of aged rats (***Fig. 2***).

### The effect of aging on glial cells

#### The effect of aging on astrocytes

Senescent cells express a senescence-associated secretory phenotype, and astrocytes exhibit age-related changes similar to the changes^[41]^. In one study, the secretory pattern of senescent astrocytes was examined using a protein expression array, and the results showed that senescent astrocytes produce multiple inflammatory cytokines; the most significant increase was observed in the IL-6 secretion by a 10-fold increase, and the production of RANTES, IL-8, and ICAM-1 also increased by more than two-fold, compared with those in unaged astrocytes^[42]^. All these inflammatory cytokines act in concert to induce local inflammatory responses.

#### The effect of aging on microglia

Iron deposition is a prominent feature of aging microglia. A recent study of rats has shown that Fe concentration is three-fold higher in microglia than in neurons^[43]^. Striatal Fe was found to be the highest in the elderly, who also showed increased neuroinflammation assessed through myoinositol, a marker of the activated microglia in the brain, measured by magnetic resonance spectroscopy^[44]^. The activated microglial cells during aging produce and release superoxide and ROS. Superoxide acts as a reducing agent and can release iron from ferritin. The superoxide-mediated iron leakage from ferritin has been shown to cause membrane lipid peroxidation. Ferroptosis, a recently described form of cell death, is defined as iron-dependent regulated necrosis caused by massive lipid peroxidation-mediated membrane damage. Iron and ROS generation are involved in the process of ferroptosis^[45]^. Therefore, it is likely that a subset of microglial cells participate in long-term iron storage to minimize iron-dependent oxidative damage. Besides, the secretion of inflammatory cytokines TNF-α and IL-1β by microglia can promote iron uptake^[46]^. These pro-inflammatory mediators have also been shown to strongly influence microglia iron transport and metabolism^[47]^.

Microglia in brain aging are characterized by dystrophic morphology, including process dedifferentiation, shortening, beadiness, spheroid formation, and cytoplasmic fragmentation^[8]^. Besides, microglia in brain aging have been found to alter their surveillance phenotype to the M1-like phenotype, releasing more pro-inflammatory factors and having less dendritic branching to contact with neurons and other cells in NVU^[48]^.

Studies of microglial gene expression in the aged mice have reported the decreased expression of cytoskeletal recombinant proteins. These studies support the idea that aging microglia have less motility^[49]^. The aging microglia in the NVU not only fail to maintain healthy neurons but also damage them by reducing phagocytosis but increasing ROS production^[50]^. Furthermore, the accumulation of DNA oxidative damage in the mitochondria of microglia during aging has also been reported. The increased intracellular ROS production activates some inflammatory signaling pathways, such as nuclear factor kappa B (NF-kB), leading to neuroinflammation during brain aging^[51]^.

### The effect of aging on vascular cells

Pericytes are important components of the NVU, However, the exact role of pericytes in brain aging remains unclear. A pronounced reduction of the coverage of vessels with pericytes has been observed in the aged brain^[52]^. Although the correlation between vessel function and vascular pericyte coverage is unknown, it has been reported that the loss of pericytes in brain aging may affect the function of capillaries^[53]^. One study has shown that the pericyte loss with aging leads to brain vascular damage by two parallel pathways: On the one hand, in the adult brain, pericytes contract blood vessel walls by regulating capillary diameter, a process during the dysfunction of pericytes may obstruct capillary blood flow, and the reduction of cerebral blood flow eventually mediates chronic perfusion stress and hypoxia. On the other hand, the breakdown of the BBB caused by loss of pericytes is associated with the accumulation of several vasotoxic and/or neurotoxic macromolecules in the brain, which ultimately leads to secondary neuronal degeneration^[54]^.

The function of ECs is closely related to mitochondria. It was found that the number of mitochondria decreased dramatically, and the function of mitochondria declined significantly during brain aging^[4]^, which is harmful to ECs. The EC changes during brain aging destroy the role of the NVU in neurovascular coupling.

Neurovascular coupling is the process by which cells coordinate to increase CBF to maintain high neuronal activity. However, if the CBF response is weakened or absent, uncoupling of nerves and vessels occurs^[55]^. One of the mechanisms how senescent ECs induce NVU disruption is through the stimulation of chronic neuroinflammatory states and the activation of cytokine-induced MMPs, and the increased MMPs and the reduction of their inhibitors (tissue inhibitors of metalloproteinase or tissue inhibitor of metalloproteinases [TIMPs]) are associated with the impaired NVU in both humans and rat models of ischaemic reperfusion injury^[56]^. MMPs play a vital role in angiogenesis and can directly induce the reduced coverage of tight junction proteins^[57–58]^. Senescent ECs may also promote neurovascular uncoupling by decreasing angiogenesis and the BDNF expression^[59]^. BDNF is a growth factor produced by neurons and ECs that promotes synaptic plasticity. In addition, the age-related decreased NO bioavailability alters sensitivity of ECs to angiogenic factors^[60]^. In summary, some changes in ECs that contribute to NVU defects have been observed in brain aging (***Table 1***).

**Table 1:** Alterations of neurovascular unit constituents during aging

NVU constituents	Alterations
Neurons	Neuronal morphological rearrangement: degenerated dendritic trees and decreased dendritic shafts with loss of various dendritic spines.
	Neurons accumulate damaged and aggregated proteins, resulting in oxidative stress: increased ROS production and decreased antioxidant capacity.
	mtDNA mutations in damaged mitochondria; Endoplasmic reticulum homeostasis is impaired; the metabolic activity of peroxisomes is lower and peroxisomes release less catalase to the cytosol.
	Neurons are unable to generate sufficient ATP to sustain brain activity.
Astrocytes	Senescent astrocytes produce multiple inflammatory cytokines, such as IL-6, RANTES, IL-8, and ICAM-1.
	Astrocytes presented a flat, senescent morphology during aging.
	Senescence astrocytes affect the aging brain though oxidative stress, proteotoxic aggregation, metabolic stress, and inflammation.
Microglia	Microglia in brain aging present dystrophic morphology, including shortening, beadiness, spheroid formation, and cytoplasmic fragmentation.
	Age-dependent switch from the alternative M2 to the classical M1 phenotype.
	Aging microglia in the NVU not only fail to maintain healthy neurons but also damage them by reducing phagocytosis and increasing ROS production.
	Iron deposition is a prominent feature of aging microglia.
Pericytes	A pronounced reduction of the coverage of vessels with pericytes is observed in the aged brain.
	Ultrastructural alterations in senescent pericytes: vesicular and lipofuscin-like inclusions, increased size of mitochondria and foamy conversion.
Endothelial cells	Reduced capacity of regeneration and newly generated defective endothelial cells are characteristic alterations in aged brain.
	The NVU disruption induced by senescent endothelial cells is through the stimulation of chronic neuroinflammatory states and activation of cytokine.
	Senescent endothelial cells may also promote neurovascular uncoupling by decreasing angiogenesis and the expression of brain-derived neurotrophic factor.
NVU: neurovascular unit; IL-6: interleukin-6; RANTES: regulated on activation, normal T cell expressed and secreted; ICAM-1: intercellular adhesion molecule-1; ROS: reactive oxygen species.	

## Neurovascular unit impairment and aging-related neurodegenerative diseases

### Aged neurovascular unit and Alzheimer's disease

One analysis showed a linear increase in the permeability of the NVUs during aging, in which by the age of 60, nearly half of the population was affected by NVU-disrupted (NVU-D; permeability in more than 5% of the brain volume)^[61]^. Some immune cell and blood-borne proteins, such as serum protein, infiltrate the brain to cause a robust brain injury response by activating related signaling pathways (*e.g.*, transforming growth factor-β (TGF-β) signaling pathway). Astrocytes are primary responders that transduce the TGF-β signaling^[62]^. In turn, the activated astrocytes release inflammatory cytokines and more TGF-β *via* a positive feedback, causing damage to the brain^[63–64]^. Another study also found that high concentrations of serum albumin accumulated in the old brain hippocampus, but not in the young^[61]^. The incidence of mild cognitive impairment (MCI) is associated with the NVU breakdown in the brain hippocampus^[65]^. In addition, as an early biomarker, brain hippocampal atrophy, especially asymmetrical atrophy, predicts the transition from cognitively normal to MCI to AD^[66]^.

Astrocytes play a central role in the pathogenesis of AD. Astrocytes are highly complex cells, and their complexity is closely related to their normal function and pathogenicity. First, astrocytes act as active components of the tripartite synapses and interact with cells within the NVU, where they are critical for the maintenance of the BBB and neurovascular coupling. Second, aged astrocytes develop hypertrophy in the form of cell proliferation, as well as astrocyte damage and atrophy. These changes may modulate the cellular effects of dementia. Astrocytes respond to both neurofibrotic tangles and plaques in AD. They may also elicit potentially neuroprotective or deleterious effects in response to the hyperphosphorylation of tau and Aβ^[67]^. In addition to the hypertrophic response, oxidative DNA damage occurs in the early stages of AD, and the unrepaired DNA damage can induce senescence or apoptosis. Glial cells in AD express apoptosis-related markers, such as p53 and CD95, and the expression of aging markers, such as β-galactosidase, can be found in astrocytes^[68]^.

Senescent microglia in the NVU have been found in both aged brains and the brains of patients with AD. They exhibit impaired neuroprotective abilities and secrete molecules that drive inflammation. The accumulation of iron leads to changes in both microglial morphology and intracellular processes, resulting in an increased endoplasmic reticulum stress and a decreased autophagy. These lead to the secretion of less insulin-degrading enzyme (IDE), one of the main enzymes associated with β-amyloid turnover, which decreases the degradation of β-amyloid in the extracellular space^[69]^. Senescent microglia also exhibit a lower phagocytic ability and secrete more inflammatory cytokines and ROS. Expression of these inflammatory cytokines is associated with further microglial damage and perpetuates AD pathology^[70]^. The depositions of tau and Aβ are important neuropathological hallmarks of AD^[71]^. Furthermore, the accumulations of extracellular Aβ and tau induce the activation of glial cells, and subsequently release proinflammatory molecules. This neuroinflammatory environment leads to the NVU damage and accelerates tau phosphorylation and Aβ formation^[72]^.

A study that included 97 AD cases classified as Braak 0–Ⅱ has shown that endothelial senescence and gene expression are associated with DNA damage and senescence. It was concluded that the damage to brain ECs occurred early in AD, which is similar to the damage to neurons and glial cells^[73]^. During brain aging, DNA damage and aging of endothelial cells may lead to the dysfunction of NVUs in some elderly people.

**Figure 3:**
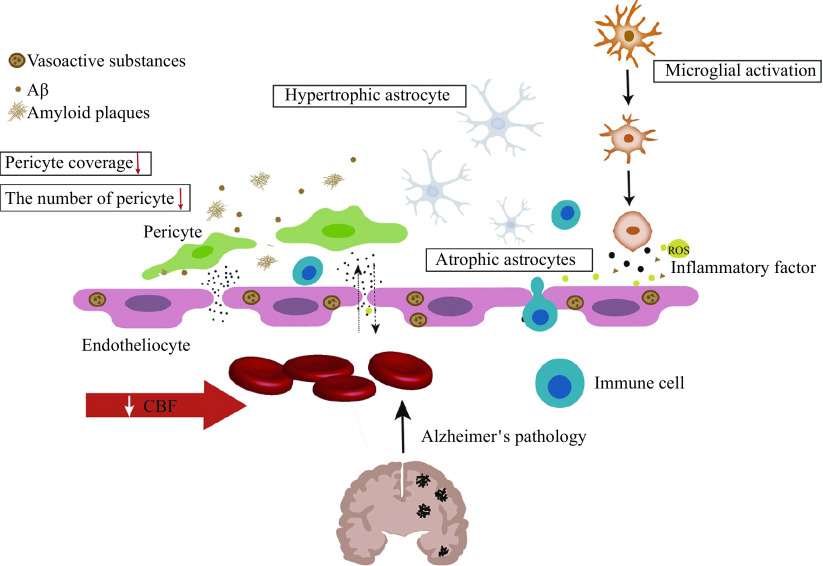
Aged neurovascular unit in the brain of Alzheimer's disease.

Pericytes directly cover the surface of ECs, and the pericytes coverage rate is defined as the percentage of ECs encircled by pericytes^[74]^. Pericytes coverage is considered to be associated with the barrier strength of the NVU^[75]^. During normal aging, the loss of pericyte coverage is a major risk factor for dementia in both rodents and humans^[76]^. However, more pericytes seem to be lost in the disease state than in normal aging. The reduction in the number of pericytes can be up to 60% in patients with AD, compared with controls^[77]^. In addition, the loss of pericytes was positively correlated with the decreased CBF. Data have shown that there is the reduced CBF in pericyte-deficient mice, especially in the white matter of these mice^[78]^. However, there is disagreement in the investigations regarding whether pericytes can directly control vasoconstriction and thus blood flow^[74]^. It is generally believed that ECs can secrete some vasoactive substances, such as endothelin-1. These vasoactive substances can bind directly to the receptors on pericytes and elicit pericyte contraction by inositol phosphate pathways^[79]^. For example, one study found an increased level of vasoactive substances in the brain of patients with AD^[80]^. During aging, ECs secrete nitric oxide to achieve vasodilation through pericyte relaxation^[81]^. The dysfunction of pericyte-endothelial crosstalk, a decreased number of pericytes, and loss of pericyte coverage contribute to a local reduction in brain perfusion. For example, aberrant angiogenesis caused by hypoperfusion has been observed in AD brain tissues^[82]^, and serves as one of the signs of AD (***Fig. 3***).

### Aged neurovascular unit and Parkinson's disease

PD is the second most common neurodegenerative disease with both motor and non-motor symptoms. There are two defined features of PD. One is the degeneration of dopaminergic neurons in the substantia nigra pars compacta (SNc). SNc dopaminergic neurons have a set of characteristics, including a long, highly branched axon, autonomous activity, and an elevated mitochondrial oxidant stress, which seem to render these neurons vulnerable to aging^[83]^. Due to their high energy needs and metabolic demands, SNc dopaminergic neurons are rich in abundant mitochondria^[84]^. Mitophagy is disturbed during aging^[85]^, while a decline in energy support leads to a reduction in mitochondria repair^[86]^. In addition, mitochondrial DNA is easily susceptible to a large amount of ROS that are mainly produced by dopamine auto-oxidation and the Fenton reaction during aging^[87]^. All of these changes contribute to dopaminergic neuronal loss and degeneration.

The accumulation of intracellular proteinaceous aggregates in neurons is another key feature of PD^[83]^. Oxidative stress in the aged SNc dopaminergic neurons facilitates the aggregation of α-synuclein^[88]^. A common feature of the most neurodegenerative diseases is a poor efficacy in clearing and recycling toxic aggregating proteins and damaged organelles due to the impaired autophagy pathways. The damaged autophagy during aging leads to the accumulation of α-synuclein and a progressive loss of dopamine neuronal structural integrity/function^[89]^. Impaired autophagy during aging is also accompanied by oxidative stress and neuroinflammation, which cause mitochondrial dysfunction and further promote the degeneration of dopaminergic neurons^[90]^. Autophagy-mediated pathways might be targeted for effective PD treatment^[91]^. Phosphorylated α-synuclein was reported to be highly enriched in cortical neurons of the aged transgenic mice overexpressing α-synuclein^[92]^. The accumulation of α-synuclein makes up Lewy bodies that are confirmed to be the hallmarks of PD^[93]^, and the accumulation of α-synuclein activates microglial cells. The activated microglia lead to a series of proinflammatory changes that are ultimately neurotoxic^[94]^. In addition, soluble α-synuclein binds to microglial cell surface receptors Toll-like receptor-2 (TLR2), TLR4, and CD11b, then activates some related inflammatory pathways, including NF-kB and mitogen-activated protein kinase, indirectly causing an increased oxidative stress. Induction of NF-KB and other classical inflammatory pathways in microglia are also responsible for the activation of astrocytes^[95]^. Intact astrocytes produce antioxidants and remove toxic molecules, such as glutamate and α-synuclein, to protect and support dopaminergic neurons^[96]^. Instead of protecting and supporting dopaminergic neurons, senescent astrocytes release cytokines that are harmful to the integrity of the NVU^[97]^. Therefore, an imbalance between the protective and damaging actions of astrocytes during aging may explain the effect of aging on PD.

Over the past two decades, various PD models have provided a greater precision in understanding the process, etiology, pathology, and underlying molecular mechanisms of PD. 1-Methyl-4-Phenyl-1,2,3,6-Tetrahydropyridine (MPTP) is currently recognized as the gold standard neurotoxin and is widely used for the induction mouse model of PD^[98]^. MPTP is converted into the toxic metabolite 1-methyl-4-phenylpyridinium (MPP+) in astrocytes of the brain, and then exocytosed to the exterior. MPP+ has a high specificity for the dopamine (DA) transporter present on the membrane of dopaminergic neurons. Therefore, MPP+ is taken up by dopaminergic neurons through dopamine transporter and accumulates inside the mitochondria. When reaching a higher level in the mitochondria of dopaminergic neurons, the accumulated MPP+ ultimately diminishes the ATP/ADP ratio and causes the generation of ROS^[99]^. However, the process can be affected by the aged astrocytes.

**Figure 4:**
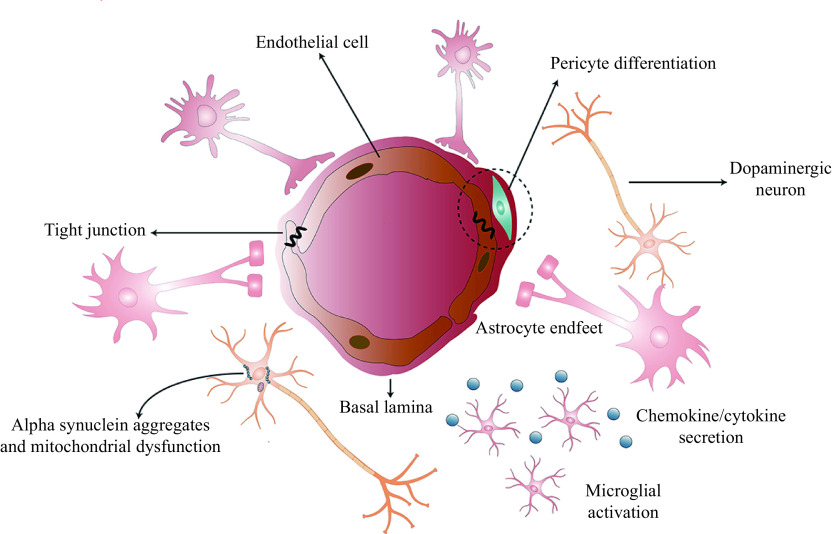
Neurovascular unit disruption in Parkinson's disease.

Some medicinal plants have numerous bioactive components, such as *Mucuna pruriens* that contain ursolic acid as one of the powerful bioactive components. Ursolic acid has shown some potent anti-oxidative and anti-inflammatory activities in MPTP-induced PD model. Chlorogenic acid found in *Withania somnifera* also shows a potent anti-Parkinsonian activity in toxin-induced PD model. *Mucuna pruriens* also shows a better neuroprotective activity and anti-apoptotic activity in the MPTP-induced Parkinsonian mouse model. Considering that *Mucuna pruriens* has a vital bioactive component responsible for its anti-PD and anti-dyskinetic activity, it may offer a vital drug for PD patients^[100]^ (***Fig. 4***).

## Novel targets for therapeutic intervention

Age-related changes in the NVU have been increasingly acknowledged as the key factors that promote vulnerability to neurodegeneration. We have not seen any immediate hope of reversing the aging process in senile NVUs, despite tremendous progress made in aging research. One of the effective ways is to counteract detrimental factors related to NVU during aging. As mentioned in the previous sections, oxidative stress and altered antioxidant systems play critical roles in NVU aging. Several studies have demonstrated that dietary interventions may regulate ROS production and oxidative damage repair. Mushrooms, known for their potent antioxidant properties, have attracted research interest for their potential neuroprotective, antioxidant, and anti-inflammatory effects and their ability to restore mitochondrial homeostasis^[101]^. Bilirubin is the final product of heme metabolism; however, this bile pigment has been endowed with a strong antioxidant activity in recent years. The cytoprotective role of bilirubin due to its scavenging activity of ROS may take place under the selected conditions and in certain organs, such as the brain^[102]^.

Hormesis is a dose-response phenomenon characterized by low-dose stimulation and high-dose inhibition, which is the core of cell adaptive responses and the origin of biological organization^[103]^. For example, a physiological amount of NO is neuroprotective, such as functioning in the regulation of synaptic plasticity, the sleep-wake cycle, and hormone secretion. However, high concentrations of NO are neurotoxic^[104]^. Ceramides mediate transient increased hormonal effects in membrane-associated oxidative stress. The pretreatment of neurons with subtoxic concentrations of ceramide results in an increased resistance of the neurons to high levels of oxidative stress subsequently^[105]^. This neuroprotective effect of the increased subtoxicity of cellular oxidative stress is known as "preconditioning"^[106]^. To adapt to changes in the NVU and different types of injuries, brain cells have evolved networks of responses that detect and control diverse forms of stress under the control of redox-dependent genes (vitagenes). Heat shock proteins and lipoxin A4 are examples of such genes. Besides, the vitagenes network consists of genes such as Nrf2-dependent enzymes heme oxygenase and γ-glutamyl cysteine ligase, which can sense oxidative damage and promote cell survival under physiopathological conditions^[107]^. Treatments targeting vitagenes are expected to be new strategies for neurodegenerative diseases in the future.

## Conclusions

Aging is a primary risk factor for most neurodegenerative diseases, including AD and PD. Age-related damage to the NVU gradually becomes a characteristic of physiological aging. Although the components of NVU cooperate with each other, aging leads to structural and functional impairments of NVU. Eventually, neurodegeneration increases with aging. While significant progress has been made in aging research, reversing the effects of aging in dysfunctional NVUs remains an enormous challenge. Maintaining an intact NVU in the brain may be a feasible way to delay and prevent the aging process.
